# Association between the type of thyroid dysfunction induced by immune checkpoint inhibitors and prognosis in cancer patients

**DOI:** 10.1186/s12902-022-01004-8

**Published:** 2022-04-04

**Authors:** Han-sang Baek, Chaiho Jeong, Kabsoo Shin, Jaejun Lee, Heysun Suh, Dong-Jun Lim, Moo Il Kang, Jeonghoon Ha

**Affiliations:** 1 Division of Endocrinology and Metabolism, Department of Internal Medicine, College of Medicine, Seoul St. Mary’s Hospital, The Catholic University of Korea, Banpo-daero, Seocho-gu, 222 Seoul, Republic of Korea; 2 Division of Endocrinology and Metabolism, Department of Internal Medicine, College of Medicine, Uijeongbu St. Mary’s Hospital, The Catholic University of Korea, Uijeongbu, Republic of Korea; 3 Division of Medical Oncology, Department of Internal Medicine, College of Medicine, Seoul St. Mary’s Hospital, The Catholic University of Korea, Seoul, Republic of Korea; 4 Department of Internal Medicines, Armed Forces Goyang Hospital, Goyang, Republic of Korea; 5 Department of Family Medicine, Gachon University College of Medicine, Incheon, Republic of Korea

**Keywords:** Immune Checkpoint Inhibitors, Hypothyroidism, Survival, Mortality, Thyroid Function Tests

## Abstract

**Background:**

Immune checkpoint inhibitors (ICIs) cause thyroid immune-related adverse effects (irAEs). However, associations between each type of thyroid immune-related adverse effect (irAE) and the anti-tumor effect of ICI remains unknown. This study aimed to determine the effects of each type of thyroid dysfunction on patient survival.

**Methods:**

Patients who initiated ICI treatment from January 2015 to December 2019 in Seoul St. Mary’s Hospital were retrospectively analyzed. Thyroid dysfunction was classified into four types: newly developed overt or subclinical hypothyroidism, thyrotoxicosis, worsened hypothyroidism, and subclinical hyperthyroidism. Patients were divided into two groups according to the presence or absence of thyroid dysfunction.

**Results:**

Among the 191 patients, 64 (33.5%) developed thyroid irAEs. There was no significant difference in age, sex, or cancer type between the two groups. The overall survival in patients with thyroid irAEs was significantly higher than that in patients without thyroid irAEs (25 months vs. 18 months, respectively, *p* = 0.005). After adjusting for confounding factors, the hazard ratio for mortality in the thyroid irAE group compared to the no thyroid irAE group was 0.480 (*p* = 0.006). Newly developed overt or subclinical hypothyroidism patients showed a significantly lower hazard ratio for mortality of 0.324 (*p* = 0.002). Patients with thyrotoxicosis showed a worse hazard ratio for mortality than those without thyroid irAE, although the difference was not statistically significant.

**Conclusions:**

It was verified that ICI treatment-induced thyroid dysfunction was associated with better survival, even in the real-world practice. Thus, endocrinologists should cooperate with oncologists to monitor patients treated with ICIs.

**Supplementary Information:**

The online version contains supplementary material available at 10.1186/s12902-022-01004-8.

## Background

Complex interactions between various types of immune cells are required to elicit an effective cytotoxic immune response against tumor cells [1–4]. Recently, a number of methods for treating malignancies by controlling the cytotoxic immune response have been reported [5–7]. Immune checkpoint inhibitors (ICIs) are immunomodulatory antibodies that are most commonly used to treat advanced malignancies. Programmed cell death 1 (PD-1) inhibitors and programmed cell death ligand 1 (PD-L1) inhibitors are the types of ICIs. They show anti-cancer effects by blocking the PD-1:PD-L1 interaction; thus, allowing T cells to induce tumor cell death [8]. PD-1 inhibitors (such as pembrolizumab and nivolumab) and PD-L1 inhibitors (such as atezolizumab and duvalumab) used in clinical fields have shown improved prognosis [9–15].

Immune-related adverse effects (irAEs) are known to affect endocrine organs, such as the pituitary, thyroid, and pancreas [16–19]. Thyroid dysfunction is especially associated with the use of PD-1 or PD-L1 inhibitors [20–23]. In a systematic review and meta-analysis of patients treated with nivolumab, pembrolizumab, or atezolizumab, the incidence of hypothyroidism was 7.0%, 3.9%, and 13.2%, and that of hyperthyroidism was 3.2%, 0.6%, and 8%, respectively [24]. Interestingly, patients with irAEs showed improved prognosis compared to those without irAEs [25]. Kotwal et al. [26] showed that patients with thyroid irAEs had longer overall survival and lower mortality, although they only focused on patients treated with PD-L1 inhibitors rather than PD-1 inhibitors.

From previous studies, it could be assumed that thyroid irAEs were associated with prognosis, and some factors were associated with thyroid irAEs [26, 27]. However, those previous studies were mostly based on clinical trials or specific populations with a focus on a particular ICI in each study. Although there are many studies on the associations between each type of thyroid irAE and ICI treatment, there is a lack of studies addressing associations between each type of thyroid irAE and prognosis. Furthermore, there is a need to investigate whether similar results can be obtained in a different population. Therefore, this study aimed to determine the association between each type of thyroid irAE induced by ICIs and survival using real-world practice data.

## Materials and methods

### Study population

**Fig. 1:**
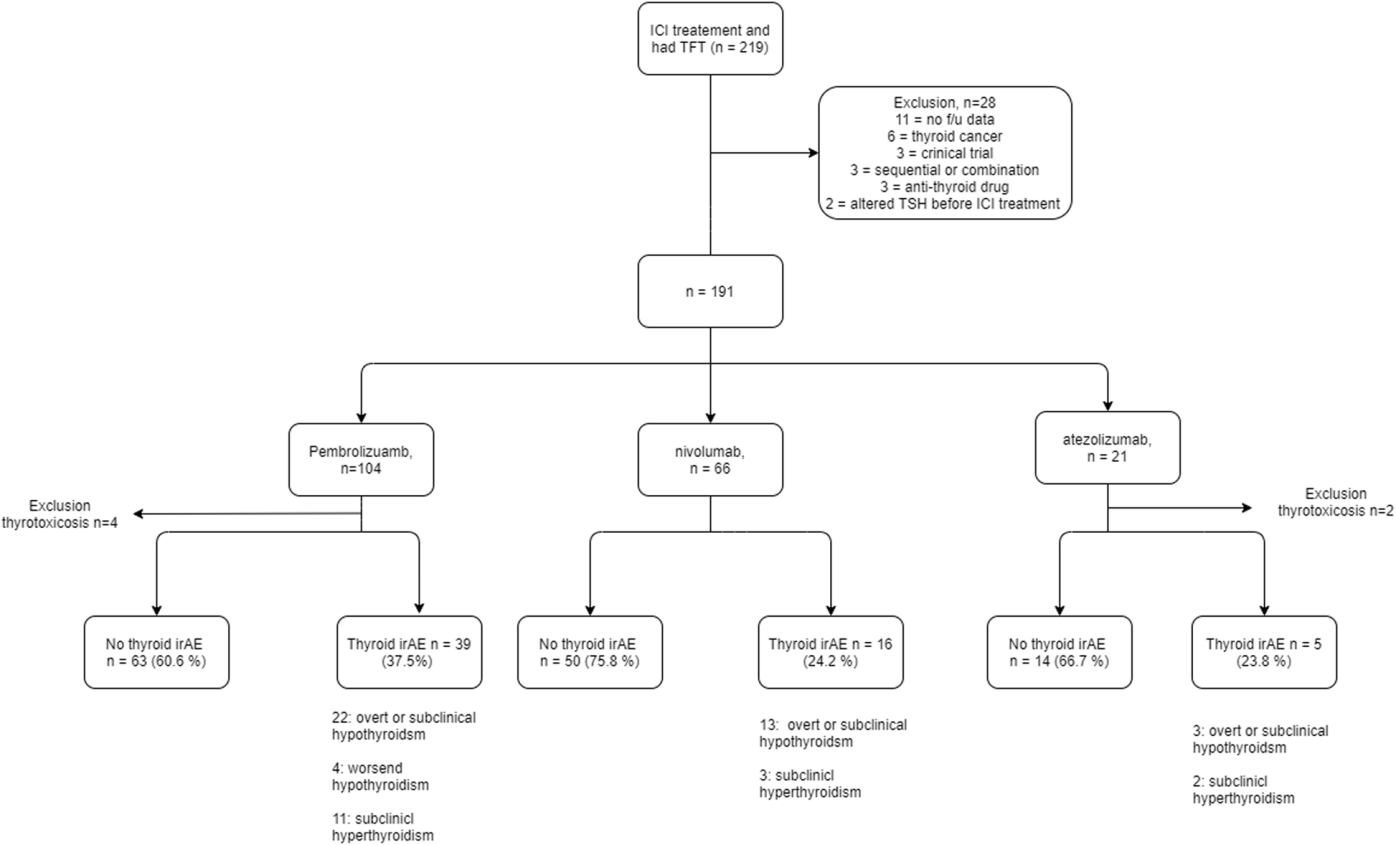
Flowchart of patient selection and data analysis showing the ratio of thyroid dysfunction by each ICI treatment. ICI, immune checkpoint inhibitor

Data of patients who had initiated PD-L1 or PD-1 treatment from January 2015 to December 2019 in Seoul St. Mary’s Hospital with thyroid function laboratory tests were retrospectively reviewed. The oncologist determined the selection of the ICIs or treatment schedules. Patients who had no follow-up data of the thyroid function test after ICI treatment initiation and those who had thyrotropin (a thyroid-stimulating hormone [TSH]) suppression treatment for thyroid cancer were excluded. Patients who underwent sequential ICI switching or combination therapy were also excluded because it was unclear which ICI could affect the result. Patients who were included in the clinical trials were also excluded because it was difficult to confirm the ICI schedule was based on medical records. Patients treated by anti-thyroid drugs for Graves’ disease or those with altered TSH before the ICI treatment initiation were also excluded. During the study period, a total of 219 patients who received ICI treatment had laboratory data for thyroid function tests. Among them, 28 patients were excluded (11 had no follow-up data, six had TSH suppression test for thyroid cancer, 3 were in clinical trials, 3 had sequential or combination ICI treatment, 3 were treated with anti-thyroid drugs and 2 had altered TSH before ICI treatment). After excluding these 28 patients, 191 patients were finally selected for the study  (Fig. 1). The study adhered to the tenets of the Declaration of Helsinki and was approved by the Institutional Review Board of Seoul St. Mary’s hospital (KC21RASI0620). Informed consent was exempted by the review board since the study was a retrospective analysis.

### Treatment definitions and classification

ICI treatment time was defined as the time from the ICI initiation date to the last ICI treatment date. Thyroid dysfunction was classified into four types: 1) newly developed overt hypothyroidism – patients who had TSH ≥ 4.8 mIU/mL and free T4 < 0.89 ng/mL; 2) thyrotoxicosis – patients who had suppressed TSH (< 0.5 mIU/mL) and increased free T4 (> 1.8 ng/mL); 3) worsened hypothyroidism – patients who had an increased dose of T4 replacement after ICI treatment; and 4) subclinical hyperthyroidism – patients who had suppressed TSH (< 0.5 mIU/mL) and normal level of free T4 (0.89 to 1.76 ng/mL). The classification was based on laboratory test done after finishing ICI treatment cycles. Some patients (12) developed hypothyroidism followed by thyrotoxicosis (6) or subclinical hyperthyroidism (6) after ICI initiation. These patients were included in overt hypothyroidism. In addition, remained thyrotoxicosis patients were excluded mainly due to the fact that the thyroid dysfunction was not corrected. It might repreemnt a bias also indicating that patients were not correctly managed. More detailed characteristics of the six patients with thyrotoxicosis are summarized in Supplementary table 1 (Fig. 1). Patients were divided into two groups: those who had any thyroid dysfunction mentioned above and those who did not have any thyroid dysfunction. After checking the medical records, no patients had worsened hyperthyroidism. Thus, thyroid dysfunction types were divided into four groups.

### Laboratory test

The thyroid function test was performed in two ways: 1) using the BECKMAN immunoradiometric assay (IMRA) kit (Immunotech, Prague, Czech Republic), and 2) using the ADIVA Centaur electrochemiluminescence immunoassay (ECLIA) kit (Siemens Healthcare Diagnostic Inc. USA). All laboratory tests were performed at St. Mary’s Hospital, Seoul, Korea. Normal ranges were as follows: TSH of 0.55 to 4.78 uIU/mL in ECLIA and of 0.17 to 4.05 IU/mL in IMRA, free T4 of 0.89 to 1.76 ng/mL in both IRMA and ECLIA, T3 of 0.6 to 1.81 ng/mL in ECLIA and 1.2 to 2.7 nmol/L in IRMA. The cutoff positivity for anti-microsomal-antibody (TPO-Ab) and thyroglobulin antibody (Tg-Ab) was 60 U/mL in both tests. Thyroid autoantibody positivity was defined when the value was above the cutoff value.

### Statistical analysis

The two groups of patients (those with any thyroid dysfunction and those who did not have any thyroid dysfunction) were compared using the t-test or chi-squared test. Fisher’s exact test was performed when the sample size was small. Kaplan–Meier curves were used to obtain overall survival using the log-rank *p* value. The Cox proportional-hazards model was used to adjust for confounding factors. SPSS v.24 (IBM Corp., New York, NY; formerly SPSS Inc., Chicago, IL, USA) was used for all statistical analyses. Graphs were produced using Prism version 8.02 (GraphPad Software Inc., La Jolla, CA, USA).

## Results

### Thyroid dysfunction and overall survival

**Fig. 2:**
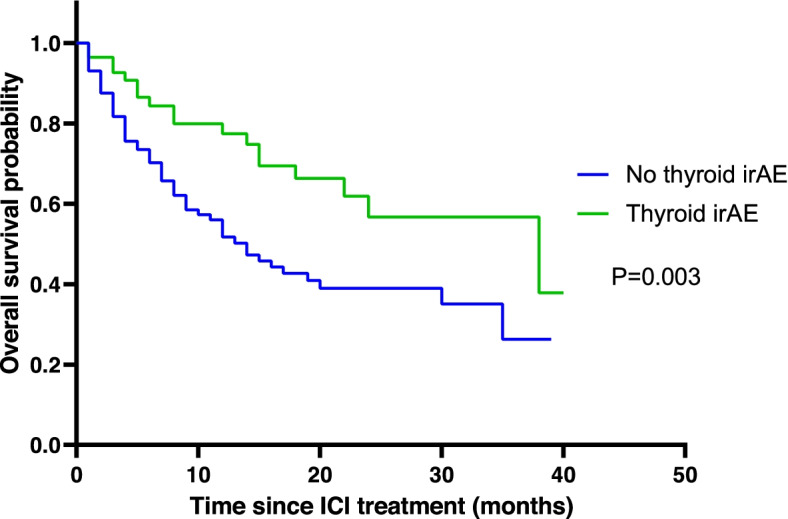
Kaplan-Meir survival curves of overall survival comparing between the thyroid irAE group and the no thyroid irAE group after ICI treatment. ICI, immune checkpoint inhibitor

Patients who received pembrolizumab (*n* = 100), nivolumab (*n* = 66), or atezolizumab (*n* = 19) were analyzed. Among these 185 patients, 58 (31.3%) developed thyroid irAEs. The median age was 66.7 ± 11.7 years for those with thyroid irAE and 64.1 ± 11.2 years (*p* = 0.140) for those who had no thyroid irAE. The most common malignancy type was lung cancer in both groups, showing no significant difference between the two groups: 40 (69.0%) in the thyroid irAE group and 72 (56.7%) in the no thyroid irAE group (*p* = 0.238). Nineteen (63.8%) patients in the thyroid irAE group and 64 (50.4%) in the no thyroid irAE group had confirmed death by medical records (*p* = 0.038). During the study period, ICIs were mostly used in the advanced stage of malignancy in our center. Despite that, ICI treatment time was longer in the thyroid irAE group (8.1 ± 8.0 months vs. 4.0 ± 6.8 months, *p* < 0.001). There was no significant difference in body mass index (BMI) between the two groups. Four patients in each group had levothyroxine replacement before the ICI treatment. (*p* = 0.440) (Table 1). The overall median follow-up duration was 17 ± 1.4 months. The median survival time was 18.0 ± 1.6 months in the no thyroid irAE group and 27.0 ± 2.3 months in the thyroid irAE group. The overall survival in patients in the thyroid irAE group was significantly higher than that in patients in the no thyroid irAE group (*p* = 0.003). Three-year survival rate was 55.7% in the thyroid irAE group and 24.6% in the no thyroid irAE group (*p* = 0.01) (Fig. 2). After adjusting for age, sex, and cancer type, the hazard ratio (HR) for mortality in the thyroid irAE group compared to that in the no irAE group was 0.417 (95% CI 0.238–0.729) (*p* = 0.002). (Table 2).

**Table 1:** Baseline characteristics of patients after immune checkpoint inhibitor treatment

	Thyroid irAE (*N* = 58)	No thyroid irAE (*N* = 127)	*P* value
Age (years) ^a^	66.7 ± 11.1	64.1 ± 11.2	0.140
Male, n (%)	44 (75.9%)	91 (71.7%)	0.675
BMI (kg/m^2^)	23.2 ± 2.9	22.2 ± 4.0	0.060
T4 replacement before ICI treatment	4 (6.9%)	4 (3.1%)	0.440
Underlying malignancy, n (%)			0.238
lung	40 (69.0%)	72 (56.7%)	
melanoma	3 (5.2%)	19 (15.0%)	
urothelial cancer	2 (3.4%)	5 (3.9%)	
breast	0 (0%)	1 (0.8%)	
colon	0 (0%)	1 (0.8%)	
esophagus	3 (5.2%)	0 (0.0%)	
HCC	1 (1.7%)	3 (2.4%)	
jejunum	0 (0%)	1 (0.8%)	
mesothelioma	1 (1.7%)	4 (3.1%)	
head and neck cancer	1 (1.7%)	8 (6.3%)	
ovary	0 (0%)	1 (0.8%)	
pancreas	1 (1.7%)	0 (0.0%)	
renal	2 (3.4%)	2 (1.6%)	
skin	0 (0%)	1 (0.8%)	
stomach	4 (6.9%)	8 (6.3%)	
thymoma	0 (0%)	1 (0.8%)	
Immune check point inhibitor, n (%)			0.196
pembrolizumab	37 (63.8%)	63 (49.6%)	
nivolumab	16 (27.6%)	50 (39.4%)	
atezolizumab	7 (8.6%)	14 (11.0%)	
Death, n (%)	19 (32.8%)	64 (50.4%)	0.038
Treatment duration ^b^ (month)	8.1 ± 8.0	4.0 ± 6.8	< 0.001
Period from initiation of ICI treatment to death (month)	14.4 ± 10.9	9.7 ± 10 .0	0.004

*irAE* immune related Adverse Event, *BMI* Body Mass Index, *HCC* Hepatocellular Carcinoma, *ICI* Immune Check point Inhibitor

^a^Age at ICI initiation

^b^the time from ICI treatment initiation date to last ICI treatment date

**Table 2:** Cox proportional-hazards model for mortality in patients treated with immune checkpoint inhibitors

Variables	Hazard ratio for mortality	*P* value
Thyroid irAE compared to no thyroid irAE	0.417 (0.238—0.729)	0.002
Types of thyroid dysfunction ^a^		
Newly developed overt hypothyroidism (*N* = 38)	0.327 (0.160—0.667)	0.002
Worsened hypothyroidism (*N* = 4)	0.599 (0.138—2.599)	0.493
Subclinical hyperthyroidism (*N* = 16)	0.607 (0.254—1.4048)	0.260
Male compared to female	0.963 (0.580—1.601)	0.886
Nivolumab ^b^	1.189 (0.724 – 1.952)	0.493
Atezolizumab ^b^	1.739 (0.719 – 4.206)	0.220

*irAE* immune related Adverse Event, *ICI* Immune Check point Inhibitor

^a^all compared to no thyroid irAE

^b^Compared to pembrolizumab

### Patient prognosis according to each type of thyroid dysfunction

**Fig. 3:**
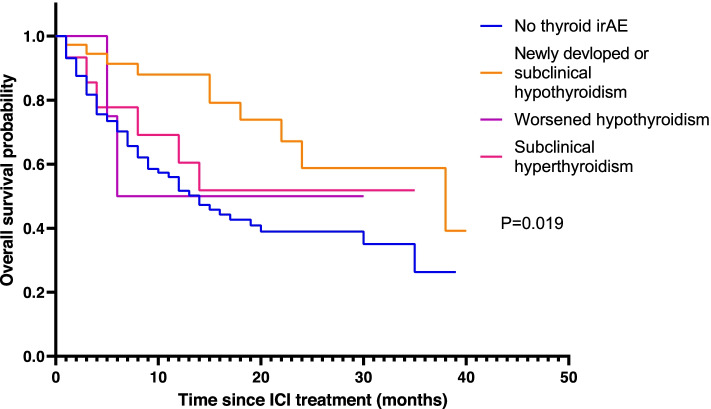
Survival curves for each thyroid dysfunction group after ICI treatment using a cox proportional-hazards model. ICI, immune checkpoint inhibitor

In subgroup analysis, the overt hypothyroidism group and the worsened hypothyroidism group showed significantly longer ICI treatment time and duration from ICI initiation to death or the last follow-up. As mentioned in Method section, because we included some patients who developed hypothyroidism after thyrotoxicosis, the period to diagnose each subgroup were different. The period to diagnose thyrotoxicosis or subclinical hyperthyroidism group was shorter than period to diagnose hypothyroidism. Of 38 overt or subclinical hypothyroidism group, 12 proceeded either from thyrotoxicosis (6) or subclinical hyperthyroidism (6) (Table 3). In subgroup analysis, the newly developed overt hypothyroidism groups showed significant improvement in prognosis (HR = 0.327 (95% CI 0.160–0.667), *p* = 0.002). The hazard ratio for mortality according to the type of ICI showed no significant differences (Table 2, Fig. 3). Patients with thyrotoxicosis showed a worse hazard ratio for mortality compared to those in the no thyroid irAE group, although the difference between the two groups was not statistically The TPO Ab positivity and the Tg-Ab positivity did not show a statistically significant difference between the thyroid irAE and no thyroid irAE groups (*p* = 0.099 for TPO-Ab and 0.591 for Tg-Ab) (Tables 4 and 5).

**Table 3:** Subgroup analysis according to the type of thyroid dysfunction

	No thyroid irAE (*N* = 127)	Thyroid irAE (*N* = 58)			
		Newly developed overt hypothyroidism (*N* = 38)	Worsened hypothyroidism (*N* = 4)	Subclinical hyperthyroidism (*N* = 16)	*P* value
Age (years) ^a^	64.1 ± 11.2	65.0 ± 12.6	70.0 ± 8.8	69.9 ± 6.3	0.191
Male, n (%)	91 (71.7%)	31 (81.6%)	1 (25%)	12 (75%)	0.1003
Death, n (%)	64 (50.4%)	11 (28.9%)	2 (50%)	6 (37.5%)	0.119
Treatment time ^b^ (month)	4.0 ± 6.8	8.9 ± 8.0	11.0 ± 7.6	4.7 ± 7.1	0.001
Period from initiation of ICI treatment to death (month)	9.7 ± 10.0	15.4 ± 10.9	15.0 ± 11.9	12.0 ± 10.9	0.025
Period from initiation of ICI treatment to diagnosis of irAE (month)	9.7 ± 10.0^c^	3.9 ± 5.3	1.0 ± 0.8	2.3 ± 5.7	< 0.001

*irAE* immune related Adverse Event, *BMI* Body Mass Index, *HCC* Hepatocellular Carcinoma, *ICI* Immune check point Inhibitor

^a^Age at ICI initiation

^b^the time from ICI treatment initiation date to last ICI treatment date

^c^It is same as period from initiation of ICI treatment to death or last follow up because thyroid irAE did not occur

**Table 4:** Association between TPO-Ab and thyroid immune related adverse effect and subgroup analysis

	Positive TPO-Ab (*N* = 7)	Negative TPO-Ab (*N* = 46)	*P*-value
Presence of thyroid irAE			0.099
Thyroid irAE	5 (71.4%)	16 (34.8%)	
No thyroid irAE	2 (28.6%)	30 (65.2%)	
Type of thyroid irAE			
Newly developed overt hypothyroidism	2 (28.6%)	12 (26.1%)	1.000
Worsened hypothyroidism	3 (42.9%)	1 (2.2%)	0.006
Subclinical hyperthyroidism	0 (0%)	3 (6.5%)	1.000

Among 196 study population, TPO-Ab were achieved in 82 patients;

*TPO-Ab* anti-microsomal-antibody, *irAE* immune related Adverse Effect

**Table 5:** Association between TG-Ab and thyroid immune related adverse effect and subgroup analysis

	Positive Tg-Ab (*N* = 4)	Negative Tg-Ab (*N* = 16)	*P*-value
Presence of thyroid irAE			0.591
Thyroid irAE	3 (75.0%)	8 (50.0%)	
No thyroid irAE	1 (25.0%)	8 (50.0%)	
Type of thyroid irAE			
Newly developed overt hypothyroidism	1 (25.0%)	4 (25.0%)	1.000
Worsened hypothyroidism	2 (50.0%)	1 (6.2%)	0.088
Subclinical hyperthyroidism	0 (0%)	3 (18.8%)	1.000

Among 196 study population, Tg-Ab were achieved in 25 patients

*Tg-Ab* Thyroglobulin-Antibody, *irAE* immune related Adverse Effect

## Discussion

In our study, the thyroid irAE group showed better prognosis than the no thyroid irAE group, regardless of age, sex, ICI, or type of underlying malignancy. In particular, the newly developed hypothyroidism group showed a significantly better prognosis.

In our study, 31.3% of patients developed thyroid irAEs. Although the occurrence rate was slightly different from those in other studies [24, 26, 28], the occurrence rate in our study was similar to that in the existing literature. The actual prevalence of thyroid dysfunction could be higher because we only included patients who underwent thyroid function tests at ICI treatment initiation. Because most practice was performed by oncologists and not endocrinologists, thyroid function tests were often omitted.

The patients in the thyroid dysfunction group, especially those with newly developed overt hypothyroidism, showed better prognosis than patients in the no thyroid irAE group. Even after adjusting for sex, age, and cancer type, this same result was consistently obtained. Similar results have been reported extensively [26, 27, 29, 30]. However, these studies either included relatively small numbers of patients or included those with only a specific cancer type or treated with a particular ICI regimen. Kotwal et al. showed improved survival in the thyroid dysfunction group, although they only included patients treated with a PD-L1 inhibitor [26]. Lima Ferreira et al. recently reported improved survival in patients with thyroid dysfunction due to several cancer types and ICI types [27]. However, none of them showed a difference in the thyroid dysfunction type. Moreover, data from Korean patients have rarely been reported.

Newly developed overt hypothyroidism patients showed significantly lower hazard ratio mortality, whereas the thyrotoxicosis group showed a high hazards ratio. In fact, previous studies shown ICI induced thyrotoxicosis would eventually develop hypothyroidism[28, 31, 32]. In our study, period from ICI initiation to diagnosis of thyrotoxicosis was shorter than period to diagnosis of hypothyroidism. In addition, among 38 hypothyroidism patients, 12 experienced thyrotoxicosis period.

On the other hand, many studies have suggested that hyperthyroidism was linked to poorer cancer prognosis [33]. However, considering that patients who had already taken a thyroid hormone for hypothyroidism showed better prognosis, the TSH stimulation pathway might be associated with prognosis. The underlying mechanisms involved in thyroid irAE and ICI treatment are not yet fully understood [25]. Thyroid irAEs often manifest as asymptomatic thyrotoxicosis, followed by a rapid transition to hypothyroidism [31]. Intrathyroidal predominance of specific T lymphocytes is thought to be associated with ICI-induced thyroiditis [34]. However, how ICI efficacy and thyroid irAEs are connected remains unknown. Previous studies have suggested that some immune pathways involving T cells or NK cells influenced thyroiditis with an anti-cancer effect [21, 22, 35]. However, the underlying mechanisms remain unknown. Moreover, the link between irAEs and anti-tumor effects remains unclear [25].

There is no reliable marker for predicting the prognosis, response, or adverse events after ICI treatment [36]. Recently, in one study in a single center in Korea, a positivity for thyroid autoantibodies could predict the progression to overt hypothyroidism [28]. In a small group study, a low frequency of thyroid autoantibody was observed, suggesting that there might be a different pathogenesis between ICI-induced thyroiditis and classical autoimmune thyroiditis [37]. In our results, TPO-Ab positivity and Tg-Ab was not significantly different between the thyroid irAE group and the no thyroid irAE group, However, in the subgroup comparison, there was a significant difference among the groups. Positive TPO-Ab was associated with levothyroxine dose elevation. This is similar to the results from a previous study [26, 28]. This suggests that thyroid autoantibodies could predict the course after ICI treatment.

Many studies have reported that a higher BMI was associated with better cancer prognosis in a variety of cancer types [38–41]. Rena et al. reported an association between higher BMI and ICI-induced thyroiditis [38]. However, in our study, BMI did not show a significant difference among patients with thyroid dysfunction. In a study by Pollack Rena et al. [38], 20% of patients had a BMI > 30. However, only 2.6% of the patients had a BMI than higher than 30 in our study. The ethnic difference might be the reason for the different results. The relation between BMI and ICI induced thyroiditis and furthermore, cancer prognosis among variant population should be investigated in the future.

This study has some limitations. First, the study population was heterogeneous due to the retrospective nature of the study. ICI treatment was performed by various oncologists; therefore, thyroid function test was not routinely performed in all cases. A well-designed prospective analysis will overcome these limitations. Second, the median follow-up period was relatively short because the use of ICIs was recently started in our center. We could not include duvalumab, another PD-L1 inhibitor, because this agent was stated to be used in our hospital in 2020 with a follow-up duration of too short. Third, the conclusions about the prognosis in the subgroups of patients affected by thyrotoxicosis and worsened hypothyroidism were hardly drawn because of the low number affected, which justifies the non-significance result obtained in the hazard ratios. Finally, we included TPO-Ab results regardless of measurement timing for before or after the ICI treatment. Because TPO-Ab positivity itself could be diagnostic of thyroid disease, it could make some bias. In addition, we could not include euthyroid state with TPO-Ab positivity suggesting another form of thyroid dysfunction because of insurance issues.

## Conclusion

It was verified that ICI treatment-induced thyroid dysfunction was associated with better survival in the real-world practice of Korean patients. The overall prognosis was the best when newly developed hypothyroidism occurred, it implies that the thyroid dysfunction maybe related to the higher sensitivity to the treatment.

## Supplementary Information

**Additional file 1:** **SupplementaryTable 1.** Characteristics of thyrotoxicosis in patients.

## Data Availability

The datasets generated and/or analysed during the current study are not publicly available due to personal data protection legislation but are available from the first author or corresponding author on reasonable request.

## Abbreviations

ICI: Immune checkpoint inhibitors
irAE: Immune-related adverse effects
TSH: Thyroid-stimulating hormone
TPO-Ab: Anti-microsomal-antibody
Tg-Ab: Thyroglobulin antibody
BMI: Body mass index
