# Supplementary material for: Association between the type of thyroid dysfunction induced by immune checkpoint inhibitors and prognosis in cancer patients
Source: BMC Endocr Disord. 2022 Apr 4;22:89. doi: 10.1186/s12902-022-01004-8 (PMC8981627; doi:10.1186/s12902-022-01004-8)
Supplement: Supplementary file 1 — Additional file 1: SupplementaryTable 1. Characteristics of thyrotoxicosis in patients. [file 12902_2022_1004_MOESM1_ESM.docx]

**Supplementary Table 1** Characteristics of thyrotoxicosis in patients

| Case | 1 | 2 | 3 | 4 | 5 | 6 |
| --- | --- | --- | --- | --- | --- | --- |
| Age/sex | 64/M | 71/M | 62/M | 62/F | 59/M | 75/F |
| Malignancy /ICI | Lung/  atezolizumab | Lung/  atezolizumab | Thymoma/  pembrolizumab | Melanoma/  pembrolizumab | Lung/  pembrolizumab | Melanoma/  pembrolizumab |
| Period from initiation of ICI treatment to death  (month) | 7 | 3 | 7 | 3 | 18 | 1 |
| Free T4 | 1.83 | 2.32 | 17.3^a^ | 7.19 | 2.17 | 2.04 |
| TSH | 0.012 | 0.011 | 0.012 | 0.041 | 0.012 | 0.019 |
| TPO-Ab | No data | No data | 373.9 | 7668.7 | No data | No data |
| Tg-Ab | No data | No data | >2500 | >2500 | No data | No data |
| TSH-R-Ab | No data | No data | < 0.3 | < 0.3 | No data | No data |
| Cessation of ICI | No cessation | Cessation d/t cancer progression | No cessation | Cessation d/t thyrotoxicosis | No cessation | Cessation d/t cancer progression and decreased performance |

^a^Total form of T4, free T4 was not available (reference range, 3.2-12.6 ug/dL)

*ICI* immune checkpoint inhibitor, *TPO-Ab* anti-microsomal-antibody, *Tg-Ab* thyroglobulin-antibody, *TSH-R-Ab* thyrotropin receptor antibody, *d/t* due to

Reference range for free T4, 0.89 - 1.76 ng/dL; for TSH, 0.55 - 4.78 uIU/mL; for TPO-Ab, <60U/mL; for Tg-Ab, <60U/mL; for TSH-R-Ab, < 1.75 IU/L
